# MiR-574-5p: A Circulating Marker of Thoracic Aortic Aneurysm

**DOI:** 10.3390/ijms20163924

**Published:** 2019-08-12

**Authors:** Adeline Boileau, Christian L. Lino Cardenas, Audrey Courtois, Lu Zhang, Rodosthenis S. Rodosthenous, Saumya Das, Natzi Sakalihasan, Jean-Baptiste Michel, Mark E. Lindsay, Yvan Devaux

**Affiliations:** 1 Cardiovascular Research Unit, Luxembourg Institute of Health, 1A-B rue Edison, L-1445 Strassen, Luxembourg; 2 Cardiovascular Research Center, Massachusetts General Hospital and Harvard Medical School, Richard B. Simches Research Center 185 Cambridge Street Suite 3201, Boston, MA 02114, USA; 3 Surgical Research Center, Department of Cardiovascular and Thoracic Surgery, University Hospital of Liege, Avenue de l’hôpital 1, 4000 Liège, Belgium; 4 UMR1148 INSERM and University Paris Diderot, 46 rue Henri-Huchard, 75877 Paris, France

**Keywords:** circulating microRNAs, thoracic aortic aneurysm, biomarker

## Abstract

Thoracic aortic aneurysm (TAA) can lead to fatal complications such as aortic dissection. Since aneurysm dimension poorly predicts dissection risk, microRNAs (miRNAs) may be useful to diagnose or risk stratify TAA patients. We aim to identify miRNAs associated with TAA pathogenesis and that are possibly able to improve TAA diagnosis. MiRNA microarray experiments of aortic media tissue samples from 19 TAA patients and 19 controls allowed identifying 232 differentially expressed miRNAs. Using interaction networks between these miRNAs and 690 genes associated with TAA, we identified miR-574-5p as a potential contributor of TAA pathogenesis. Interestingly, miR-574-5p was significantly down-regulated in the TAA tissue compared to the controls, but was up-regulated in serum samples from a separate group of 28 TAA patients compared to 20 controls (*p* < 0.001). MiR-574-5p serum levels discriminated TAA patients from controls with an area under the receiver operating characteristic curve of 0.87. In the *Fbn1^C1041G/+^* mouse model, miR-574-5p was down-regulated in aortic tissue compared to wild-type (*p* < 0.05), and up-regulated in plasma extracellular vesicles from *Fbn1^C1041G/+^* mice compared to wild-type mice (*p* < 0.05). Furthermore, in vascular smooth muscle cells, angiotensin II appears to induce miR-574-5p secretion in extracellular vesicles. In conclusion, miR-574-5p is associated with TAA pathogenesis and may help in diagnosing this disease.

## 1. Introduction

Thoracic aortic aneurysm (TAA) is a life-threatening disease with an incidence of approximately 10/100,000 population [1]. TAA is characterized by an asymptomatic enlargement of the thoracic aorta. Patients with TAA are at higher risk to develop severe aortic complications as intramural hematoma, dissection, or rupture of the aorta. To prevent such complications, patients require regular follow-up and sometimes prophylactic aortic surgery [2]. TAA is a silent disease often fortuitously diagnosed through medical imaging tests prescribed in the context of another disease or during routine cardiovascular exams, thus novel targeted diagnostic tools are needed to identify high-risk patients who will benefit from tailored healthcare [3].

TAAs have different etiologies. Whereas monogenic etiologies are often observed in young patients [4] as for instance Marfan syndrome caused by mutation of the *FBN1* gene encoding the protein fibrillin-1 [5] or Loeys-Dietz syndrome, due to mutations in TGF-β related genes such as *TGFBR1* or *TGFBR2* amongst others, older patients may show a degenerative form of aneurysm and the presence of a bicuspid aortic valve also promotes TAA [6]. Regardless of etiology, similar histological features are observed in the aneurysmal aortic wall: Disorganization of extracellular matrix [7] and loss and phenotypic modification of vascular smooth muscle cells (VSMCs) [8]. In TAA, VSMCs are subject to a phenotypic switch, from a contractile to a secretory phenotype characterized by an increase of secretory, migratory, and proliferation capacities, with decreased contractility [9,10]. Although monogenic alterations have been identified that induce or foster TAA, no pathogenic variant can be identified for 70% of patients with familial TAA [11]. Physiological mechanisms such as the renin-angiotensin system and the TGF-β pathway have also been implicated in TAA [12] and in VSMC phenotypic switch [13]. To decipher TAA mechanisms, several animal models were developed, harboring mutations responsible for familial or syndromic forms of TAA, such as the *Fbn1^C1041G/+^* mice model that mimics the Marfan syndrome [14]. However, the high number of genes involved and the numerous mechanisms thought to be involved in TAA formation and progression support the complexity of this disease, which is yet to be fully understood [15].

MicroRNAs (miRNAs) are small non-coding RNA molecules, between 18 and 22 nucleotides of length, obtained after processing of their precursor or pre-miRNA. Through their seed sequence, miRNAs bind to the 3’ untranslated region of target messenger RNAs, thereby inhibiting their translation into proteins [16]. A single miRNA can target several messenger RNAs, and miRNAs are considered as post-transcriptional regulators that are able to actively participate in physiological and pathological processes [17]. In the context of TAA, expression profiles of miRNAs are altered in the aortic media of patients [18] and in the aorta from *Fbn1^C1041G/+^* mice [19], suggesting possible involvement in TAA pathogenesis. MiRNAs are also implicated in specific features of TAA. For instance, miR-143 and miR-145 induce VSMC differentiation towards a contractile phenotype [20].

MiRNAs are also relatively stable and easily accessible in blood (termed circulating or extracellular miRNAs), thereby conferring potential as diagnostic or prognostic biomarkers. The presence of miRNAs in the blood can be attributed either to a passive leakage from damaged cells or to active secretion [21]. In the latter case, miRNAs are associated with RNA-binding proteins [22], high-density lipoproteins [23]; or are encapsulated in extracellular vesicles (EVs) [24]. The association between circulating miRNAs and acute aortic dissection has recently been reported [25]. Furthermore, signatures of circulating miRNAs have been identified in the bicuspid aortic valve disease [26]. These reports support the use of circulating miRNAs as diagnostic biomarkers of TAA, a possibility that we addressed in the present study using both tissue and liquid biopsies from TAA patients and *Fbn1^C1041G/+^* mice.

## 2. Results

### 2.1. Study Design

The design of this study is presented in Figure S1 and is divided in two major axes. The first axis aims to discover miRNA candidates and to evaluate their biomarker ability in human samples. The second axis proposes a functional characterization in the *Fbn1^C1041G/+^* mice model.

### 2.2. Patient Characteristics

The “tissue” and “serum” cohorts were composed of TAA patients and controls without aortic aneurysm. Characteristics of these two cohorts are reported in Tables S1 and S2. In the tissue cohort, the TAA group and the control group were comparable in terms of age and sex. Among the TAA patients, two had an aortic dissection, nine had a bicuspid aortic valve, two had the Marfan syndrome, two had degenerative disease and none had the Ehler-Danlos syndrome. In the serum cohort, controls were older than the TAA patients. Three TAA patients had an aortic dissection, six had a bicuspid aortic valve, one had the Ehler-Danlos syndrome (type IV) and none had the Marfan syndrome or degenerative disease. There were more patients with hyperlipidemia and more current and former smokers in the TAA group than in the control group. Medications were not significantly different between controls and TAA patients. Table S3 compares the characteristics of patients from the serum and tissue cohorts. There were no differences of sex, aortic diameter and number of patients with aortic dissection or other aneurysm aetiology. However, patients in the serum cohort were older. Furthermore, the smoking and medication status appeared to be different between the two cohorts presumably because of the high number of missing data in the tissue cohort.

### 2.3. MiRNA Expression Profiles in Human Aortic Media

**Figure 1:**
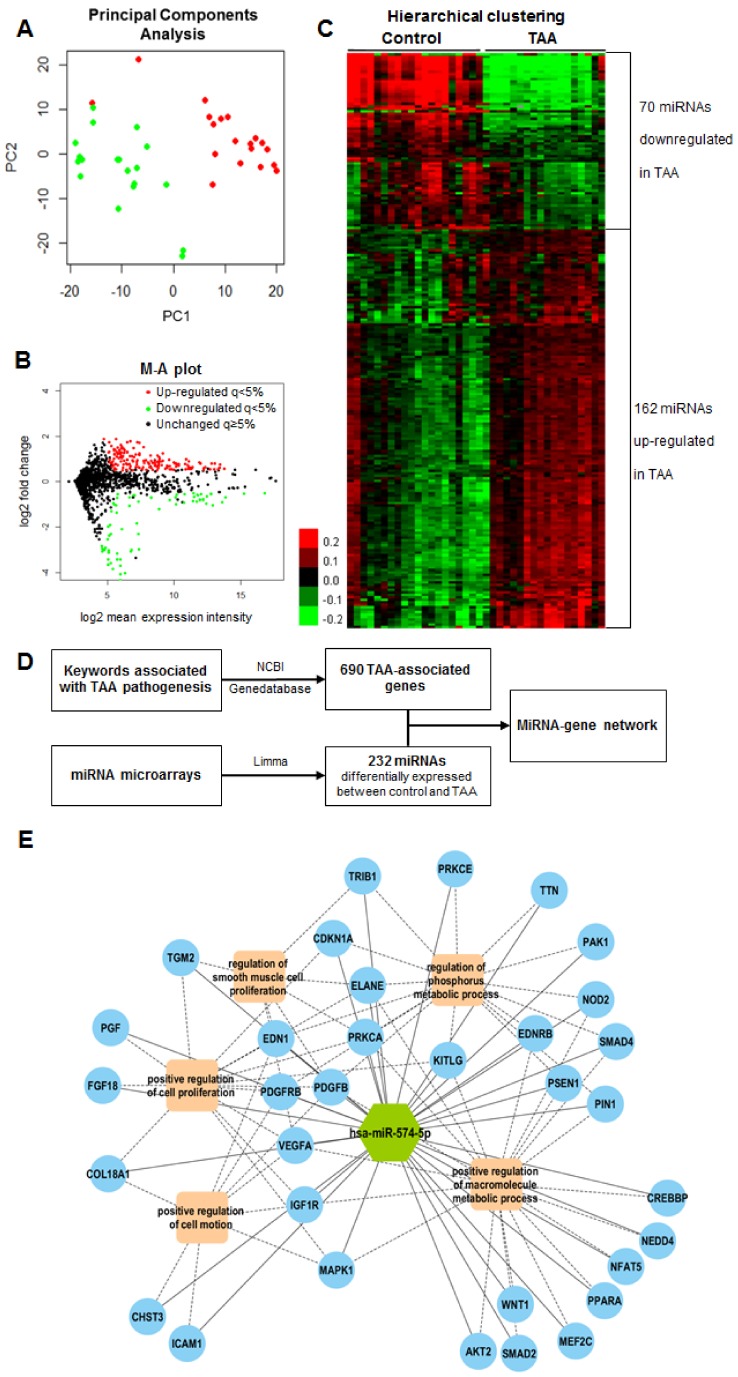
MicroRNAs (MiRNA) expression profiles in aortic tissues from the tissue cohort and miRNA-gene and protein-protein interaction network. (**A**–**C**). Microarrays were conducted on RNA samples isolated from the aortic media of 19 thoracic aortic aneurysm (TAA) patients and 19 non-aneurysmal control donors. (**A**) The principal component analysis showing the separation of TAA and control samples according to miRNA expression. (**B**) M-A plot showing the distribution of miRNAs in TAA versus control samples. Log2 transformed-fold change is shown in the vertical axis and the average signal of each miRNA is shown in the horizontal axis. (**C**) Heat-map displaying modulated miRNAs between TAA and control samples. A log2-transformed fold change > 0.5 or < −0.5, a q-value <5% and a relative expression level > 5 were used as cut-offs. Red color corresponds to miRNAs up-regulated in TAA patients and green color corresponds to miRNAs down-regulated in TAA patients compared to controls. (**D**,**E**) MiRNA-gene and protein-protein interaction network. (**D**) Procedure followed to create the interaction network. 690 TAA-associated genes were identified using the National Center for Biotechnology Information (NCBI) Gene database and literature search. 232 miRNAs were found to be differentially expressed between aneurysmal and non-aneurysmal aortic tissue using microarrays. Interactions between TAA-associated genes and differentially expressed miRNAs were predicted using TargetScan, microRNA Target Prediction Database (miRDB) and DIANA microT-CDS databases. Only the interactions predicted by at least two databases were considered. Functional protein-protein interactions identified in the IntAct database were also included in the network. (**E**) MiR-574-5p-gene interaction network. The network shows the association between-miR-574-5p, its predicted gene targets, and the top five biological processes enriched by these targets. Solid line: Interaction between miRNA and the target gene. Dash line: Association between gene targets and biological processes.

Total RNA was extracted from aortic media of 19 TAA patients and 19 controls of the tissue cohort and was hybridized onto miRNA microarrays. 473 miRNAs were detected in at least half samples and had expression levels above the expression of negative controls. The principal component analysis showed that aneurysm and control groups could be almost completely separated by miRNAs expression profiles (Figure 1A). We identified 232 differentially expressed miRNAs with an adjusted q-value < 0.05, a log2-transformed fold change > 0.5 or < −0.5, and a relative expression level > 5. As shown in Figure 1B,C, 162 miRNAs were found to be up-regulated and 70 were down-regulated in aneurysmal tissues compared to non-aneurysmal tissues.

### 2.4. Candidate MiRNA Selection

To identify miRNAs with a potential functional role in TAA, we used a network-based approach (Figure 1D). First, the literature search and National Center for Biotechnology Information (NCBI) Gene database allowed the identification of 690 genes presumably associated with TAA (Table S4). Second, we interrogated the targetScan, microRNA Target Prediction Database (miRDB) and DIANA microT-CDS databases to predict interactions between the 232 miRNAs identified as differentially expressed between the control and TAA in aortic media using microarrays. 5510 interactions were predicted by at least two databases. Third, we interrogated the IntAct database and we identified 1072 protein-protein interactions between the 690 TAA-associated proteins. A network combining both the 5510 interactions between the 232 differentially expressed miRNAs and the 690 TAA-associated genes, and the 1072 interactions between the 690 TAA-associated proteins, is displayed in Figure S2. The network was divided into eight modules according to the density of links between miRNAs and genes. Biological processes enriched by genes of each module are presented in Table S5, to the exception of modules two and eight which included only four and three genes, respectively. Interestingly, module six was enriched by biological processes related to vessel biology such as vasculature development and blood vessel morphogenesis. Module six included 49 miRNAs. The traffic score of each miRNA, shown in Table S6 attests for its betweenness centrality. A high traffic score indicates a high level of interaction with genes. Among the 10 miRNAs with the highest traffic scores in module six, miR-574-5p was the most differentially expressed between the aneurysmal and non-aneurysmal tissue in the microarray analysis (adjusted *p*-value 2.79E-06; Table S7). We therefore focused on miR-574-5p in subsequent analyses.

To gain insights into the potential role of miR-574-5p in TAA pathogenesis, we searched among the 690 genes previously identified as associated with TAA, which ones were predicted to be targeted by miR-574-5p. 79 genes satisfied this criterion (Table S8). We then investigated the biological processes enriched by these 79 genes. The top five most significant and non-redundant biological processes were: Regulation of phosphorus metabolic process, positive regulation of cell motion, positive regulation of macromolecule metabolic process, regulation of smooth muscle cell proliferation and positive regulation of cell proliferation. These five biological processes were enriched by 33 of the 79 genes targeted by miR-574-5p. A network illustrating these interactions is shown in Figure 1E.

### 2.5. MiR-574-5p Expression in the Human Tissue Cohort

**Figure 2:**
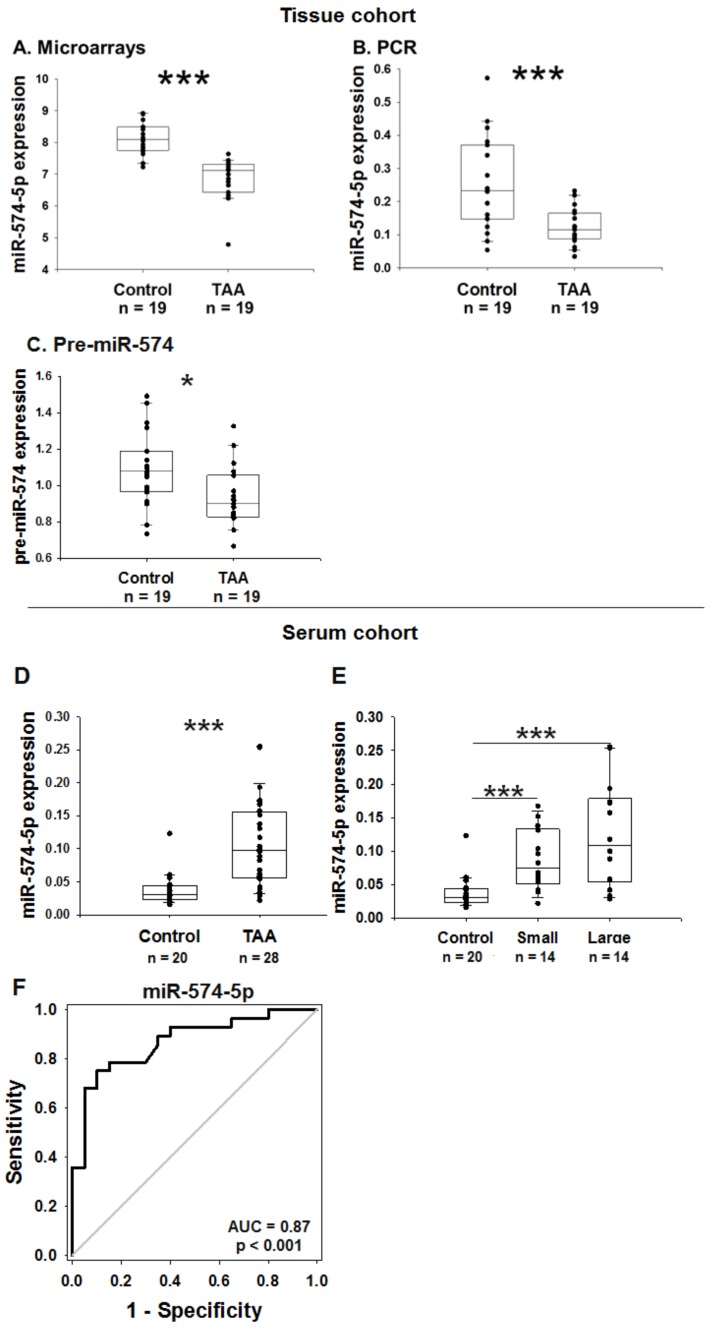
MiR-574-5p and pre-miR-574 expression in aortic tissues from the tissue cohort (**A**–**C**); and the association between serum levels of miR-574-5p and TAA in the serum cohort (**D**–**F**). (**A**) Microarray data. (**B**,**C**) Quantitative PCR data. * *p* < 0.05, *** *p* < 0.001 versus control. (**D**) Expression of miR-574-5p in the serum of TAA and control patients from the serum cohort. (**E**) Expression of miR-574-5p according to the size of the aneurysm. The small group includes patients with an aneurysm < 49 mm and the large group includes patients with an aneurysm > 49 mm. TAA: Thoracic aortic aneurysm. *** *p* < 0.001. (**F**) ROC (receiver operating characteristic) curve analysis showing the association between serum levels of miR-574-5p and TAA. AUC: Area under the curve.

Figure 2A displays the pattern of expression of miR-574-5p in the aneurysmal and non-aneurysmal aortic tissues assessed by microarrays. MiR-574-5p was significantly down-regulated in the aneurysmal group. This result was confirmed using a quantitative PCR (Figure 2B). Interestingly, pre-miR-574-5p was also down-regulated in the aneurysmal group (Figure 2C). In the aortic aneurysmal aortic tissue from different etiologies, miR-574-5p is down-regulated compared to the healthy aorta. However, miR-574-5p seems slightly up-regulated in patients with aortic dissection compared to the other etiologies (Figure S3).

### 2.6. MiR-574-5p Expression in the Human Serum Cohort

To address a potential diagnostic value of miR-574-5p, we measured its expression in serum samples from TAA patients and controls (serum cohort). Interestingly, miR-574-5p was significantly up-regulated in the serum of patients with TAA compared to the control (3-fold, *p* < 0.001; Figure 2D) and this up-regulation was higher in patients with a large (above 49 mm) aneurysm (Figure 2E). Of note, the cut-off of 49 mm corresponds to the median of the aortic diameter of the TAA group. Circulating levels of miR-574-5p are up-regulated in patients suffering TAA independently of the etiology of TAA, compared to the controls (Figure S3), and miR-574-5p levels were not significantly modulated by hyperlipidaemia or the smoking status, either in the control or in the TAA groups (Figure S4). The ROC (receiver operating characteristic) curve analysis revealed an association between miR-574-5p and the diagnostic of TAA with an area under the curve of 0.87 (Figure 2F). MiR-574-5p discriminated the TAA patients from the controls with a specificity of 85% and a sensitivity of 78.6%. These data support a diagnostic potential of miR-574-5p for TAA.

### 2.7. MiR-574-5p in Fbn1^C1041G/+^ Aortic Tissues and EVs

**Figure 3:**
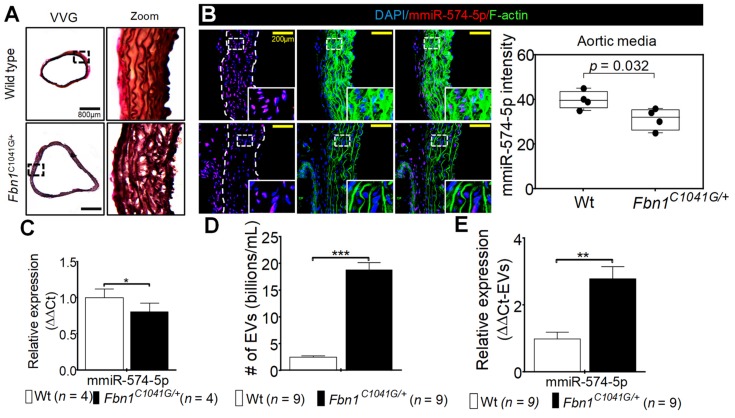
Expression of miR-574-5p in the aorta of *Fbn1^C1041G/+^* and wild-type (Wt) mice. (**A**) Staining of elastin fibers (VVG) in the aorta from Wt and *Fbn1^C1041G/+^* mice, scale bar represents 800 µm. (**B**) Left panel; immunofluorescence and fluorescent in-situ hybridization staining of miR-574-5p (red), F-actin (green) and nucleus (blue) in the section of the ascending aortic tissue from Wt (*n* = 4, two males and two females) and *Fbn1^C1041G/+^* (*n* = 4, two males and two females) mice; the yellow scale bar corresponds to 200 µm. Right panel; relative fluorescence intensity quantification of miR-574-5p (red) in the aortic media from Wt (*n* = 4, two males and two females) and *Fbn1^C1041G/+^* (*n* = 4, two males and two females) mice. (**C**) Relative expression of miR-574-5p measured using PCR in the aortic tissue from Wt (*n* = 4, two males and two females) and *Fbn1^C1041G/+^* (*n* = 4, two males and two females) mice. (**D**) Quantification of circulating extracellular vesicles (EVs) from Wt (*n* = 9, five males and four females) and *Fbn1^C1041G/+^* (*n* = 9, five males and four females) mice. (**E**) Relative expression of miR-574-5p measured in EVs from Wt (*n* = 9, five males and four females) and *Fbn1^C1041G/+^* (*n* = 9, five males and four females) mice using PCR. * *p* < 0.05; ** *p* < 0.01; *** *p* < 0.001.

To investigate miR-574-5p expression, we used the *Fbn1^C1041G/+^* mice model which is characterized by a mutation on the *Fbn1* gene [14]. Alteration of the *Fbn1* gene was effective, since elastin disorganization was present in the aortic wall of *Fbn1^C1041G/+^* mice (Figure 3A). Using fluorescence in situ hybridization (FISH) in the aortic wall, miR-574-5p appeared to be mainly localized in the nucleus of the cells, and was less expressed in aneurysmal than in normal aorta (*p* = 0.032; Figure 3B,C). These data are consistent with our previous observations in human aortic tissue (Figure 2A,B).

To understand under which form miR-574-5p navigates in the blood flow, we studied EVs in the blood of Wt and *Fbn1^C1041G/+^* mice. There was approximatively 4-fold more EVs in plasma from *Fbn1^C1041G/+^* mice compared to normal mice (*p* < 0.001; Figure 3D). Furthermore, miR-574-5p was 3-fold increased in the cargo of EVs from the plasma of *Fbn1^C1041G/+^* than from the Wt mice (*p* < 0.01; Figure 3E).

### 2.8. MiR-574-5p Expression in Murine Organs

**Figure 4:**
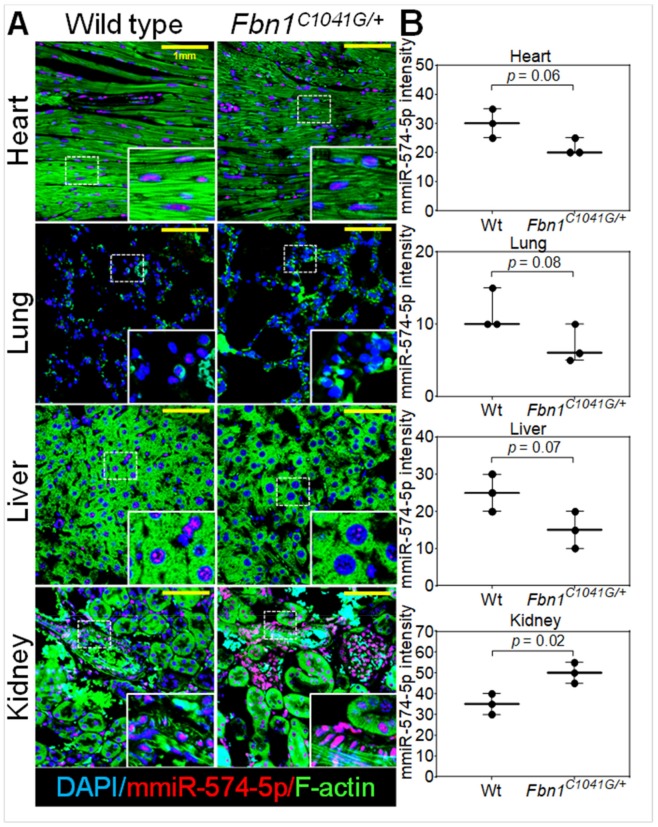
Expression of miR-574-5p in different organs (heart, lung, liver and kidney) in the wild-type (Wt) and *Fbn1^C1041G/+^* mice. (**A**) Immunofluorescence and Fluorescence in situ hybridization (FISH) of miR-574-5p (red), F-actin (green) and nucleus (blue) from Wt and *Fbn1^C1041G/+^* mice. (**B**) Relative fluorescence intensity quantification from Wt (*n* = 3, two males and one female) and *Fbn1^C1041G/+^* (*n* = 3, one male and two females) mice. The yellow scale bar corresponds to 1 mm.

The global expression of miR-574-5p was examined using FISH in several organs from the Wt and *Fbn1^C1041G/+^* mice. Levels of miR-574-5p were low in the lung and were high in the kidney (Figure 4). As previously observed in the aortic tissue, miR-574-5p was localized in the nucleus. MiR-574-5p appears to be slightly less expressed in the heart, lung and liver from the *Fbn1^C1041G/+^* mice than from the Wt mice (non-significant; Figure 4B). Conversely, miR-574-5p was highly expressed in the *Fbn1^C1041G/+^* kidney compared to the normal kidney (1.4 fold; *p* = 0.02; Figure 4B).

### 2.9. MiR-574-5p in EVs and VSMCs

**Figure 5:**
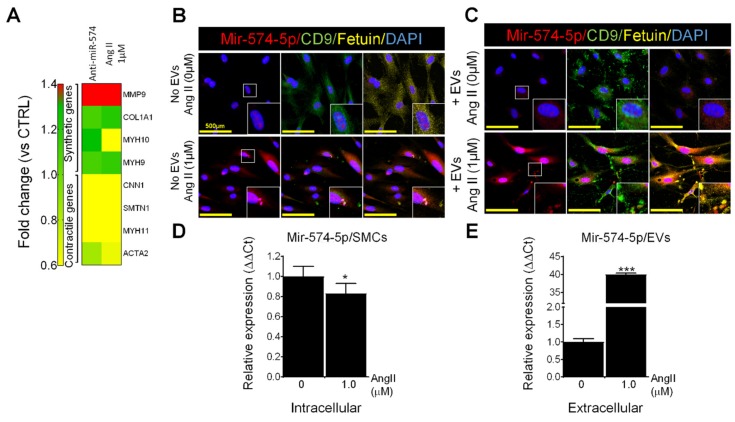
Effect of angiotensin II (Ang II) on miR-574-5p expression and EV secretion in human primary vascular smooth cells (VSMCs). (**A**) Relative expression of genes associated with synthetic or contractile phenotype measured by PCR in human VSMCs treated with anti-miR-574-5p or with Ang II (1 µM). (**B**) VSMCs treated or not with 1 µM of Ang II. (**C**) VSMCs treated with EV from normal VSMC or from VSMCs treated with 1 µM of Ang II. (**D**) Relative expression of miR-574-5p measured using PCR in VSMCs treated or not with Ang II. (**E**) Relative expression of miR-574-5p measured using PCR in EVs secreted by VSMCs treated or not with Ang II. Immunofluorescence and FISH staining of miR-574-5p (red); CD9, a membrane marker of EVs (green); fetuin, a marker of EV secreted from VSMC (yellow) and nucleus (blue) were used on primary human VSMCs. The yellow bar corresponds to 500 µm. Cells originate from an adult men. * *p* < 0.05; *** *p* < 0.001.

In cultured primary VSMCs isolated from the Wt mice, miR-574-5p was solely expressed in the nucleus and the CD63 positive extracellular staining consistent with EV-staining was only detected at low levels (Figure S5, upper panel). After treatment with EVs isolated from the plasma of the Wt mice, miR-574-5p was detected both in the nucleus and in the cytoplasm of VSMCs and several CD63-positive EVs appeared (Figure S5, middle panel). VSMCs treated with EVs from *Fbn1^C1041G/+^* mice showed higher levels of miR-574-5p (nuclear and cytoplasmic) and increased EVs formation compared to VSMCs treated with EVs from the Wt mice (Figure S5, lower panel). Furthermore, in this condition, miR-574-5p staining co-localized with several EVs (yellow staining, Figure S5 lower panel). These results suggest that intercellular communication mediated by EVs from aneurysmal syndromes may both stimulate cytoplasmic translocation of miR-574-5p and possibly mediate their packaging and excretion in EVs. It is also possible that miR-574-5p may be directly transferred to VSMCs from the EVs obtained from the *Fbn1^C1041G/+^* mice (where extracellular miR-574-5p is increased). The inhibition of miR-574-5p in human VSMC induces an up-regulation of synthetic genes such as *MMP9* and a down-regulation of “contractile genes” (Figure 5A).

### 2.10. Effects of Ang II on MiR-574-5p and EVs in Human VSMCs

In cultured primary VSMCs from healthy controls, miR-574-5p was solely present in the nucleus and CD9 and fetuin EV markers were poorly expressed. After treatment with 1 µM of Ang II for 24 h, miR-574-5p was expressed in vesicles in the cells, which were also harboring CD9 and fetuin, suggesting that these vesicles might be released as EVs (Figure 5B). Healthy human VSMC were treated with EVs from the normal human VSMC or with EVs secreted by VSMC treated with Ang II (Figure 5C). EVs from Ang II-treated cells appeared to induce an increased secretion of EVs containing miR-574-5p by VSMC. VSMC treated with Ang II had lower intracellular levels of miR-574-5p but an increased expression of this miRNA in EVs compared to untreated cells (Figure 5D,E). Furthermore, cells treated with Ang II increased the expression of MMP9 which is a secreted enzyme and decreased the expression of ACTA2 which is a cellular contractile protein (Figure 5A).

### 2.11. MiR-574-5p Expression in Mice Plasma

**Figure 6:**
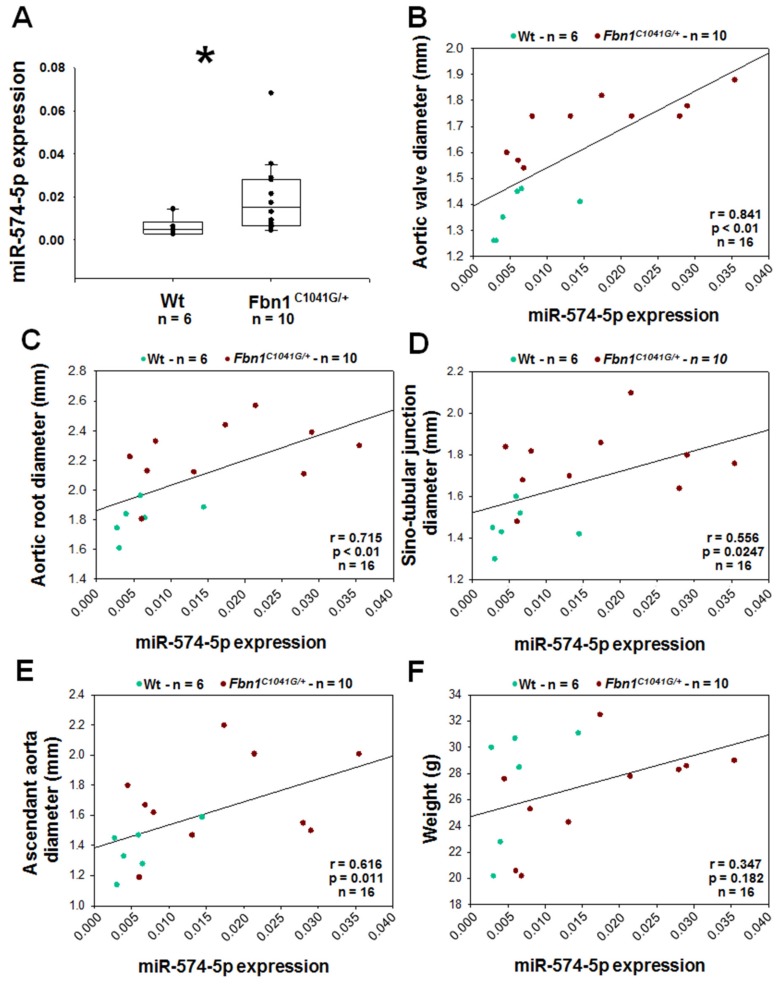
Association between plasma levels of miR-574-5p and aortic dilatation in *Fbn1^C1041G/+^* mice. (**A**) Expression of miR-574-5p in the plasma of *Fbn1^C1041G/+^* and wild-type (Wt) mice. (**B**–**F**) Spearman correlation between miR-574-5p and, respectively, the aortic valve diameter (**B**), aortic root diameter (**C**), sino-tubular junction diameter (**D**), ascendant aorta diameter (**E**) and weight (**F**). R= correlation coefficient; * *p* < 0.05; among the 16 mice, six are females (two Wt and four *Fbn1^C1041G/+^*).

MiR-574-5p expression was measured in the plasma from six Wt and 10 *Fbn1^C1041G/+^* mice. The aortic size of these mice was checked using ultrasounds (Figure S6). MiR-574-5p was over-expressed in the plasma of *Fbn1^C1041G/+^* mice versus Wt (3-fold; *p* < 0.05; Figure 6A). Furthermore, miR-574-5p expression was positively correlated with the diameter of the different segment of the aorta, respectively, the aortic valve, the aortic root, the sinotubular junction and the ascendant aorta (Figure 6B,E). Levels of miR-574-5p were not correlated with the weight of the mice (Figure 6F).

## 3. Discussion

In this study, we sought to address the potential of miRNAs as biomarkers of TAA. We used two modest-sized independent cohorts of TAA patients and controls, a first one to study miRNAs in the aortic tissue and a second to study miRNAs in serum samples. Using microarrays, we confirmed that miRNA expression profiles are altered in aneurysmal compared to the normal aortic tissue. A network-based approach incited us to focus our attention on a particular miRNA, miR-574-5p. A bioinformatics analyses supported a role for miR-574-5p in the TAA pathogenesis. Serum levels of miR-574-5p were able to discriminate TAA patients from controls, supporting the potential of this miRNA to be used as a diagnostic biomarker of TAA. Then, we investigated miR-574-5p in the Marfan *Fbn1^C1041G/+^* mouse model. We noticed that miR-574-5p expression levels were lower in the aortic tissue from the *Fbn1^C1041G/+^* mice compared to the Wt mice, while plasma levels of miR-574-5p were higher in the *Fbn1^C1041G/+^* mice compared to the Wt mice. Additionally, plasma levels of miR-574-5p were correlated with the size of the thoracic aorta. In vitro, murine VSMCs treated with EVs obtained from the plasma of the *Fbn1^C1041G/+^* mice led to higher cellular (particularly cytoplasmic) levels of miR-574-5p. Interestingly, our in vitro experiments in human VSMCs treated with Ang II are consistent with a translocation of nuclear miR-574-5p towards the cytoplasm, shipped into intracellular vesicles which may be released as EVs.

The reported incidence of TAA is probably under-estimated because of the silent evolution of the disease [3]. The real burden of the disease appears difficult to quantify for the same reasons. However, TAA is a life-threatening disease, responsible for high-risk of severe thoracic aortic complications [27]. Indeed, patients with a thoracic aortic diameter above 4.5 cm have a more than 6000-fold higher risk of aortic dissection compared to patients with small aortas (< 3.4 cm of diameter) [28]. Appropriate aortic management with a close follow-up of the aneurysm size evolution, appropriate medication and prophylactic surgery when needed all contribute to prevent the occurrence of such devastating aortic complications [27]. Prophylactic surgical intervention is currently performed only when the risk of aortic dissection exceeds the presumed operative risk. However many aortic dissections occur at aortic diameters below these thresholds [29], arguing that we currently need additional ways to stratify the risk of the aortic dissection beyond simply measuring the aortic diameter. Circulating biomarkers, including miRNAs, may have the potential to inform the risk of the aortic dissection. A study reported a specific signature of circulating miRNAs in the bicuspid aortic valve disease [26]. Interestingly, in this study, plasma levels of miR-718 were correlated with the aortic dilation, however the prediction value and the cellular origin of miR-718 have not been reported.

The approach used in the present study to isolate disease-associated miRNAs has previously shown effectiveness in other pathological contexts [30]. We combined both high-throughput profiling of miRNAs expression with protein-gene-miRNAs interaction networks. This approach is more prone to highlight miRNAs functionally associated with the disease as compared to the one-step differential expression analysis. This is critical from a biomarker prospective when we aim to identify targets functionally associated with the disease and not only mere bystanders. Dysregulated miRNAs which contribute to disease development and progression may not only be used as biomarkers but may also constitute potential therapeutic targets. Although we report an association between miR-574-5p and the diagnosis of TAA, addressing the therapeutic value of this miRNA for the aortic aneurysm is beyond the scope of this study.

Bioinformatics tools based on predicted gene targets of miR-574-5p and biological pathways enriched by these genes suggested that this miRNA might be involved in the “regulation of smooth muscle cell proliferation”, “positive regulation of cellular proliferation” and “positive regulation of cell motion”. These predictions are consistent with the increased proliferation and migration of VSMCs characterizing their phenotypic switch from contractile toward a secretory state [9], which has been observed in aortic aneurysmal media [10]. In vitro, anti-miR-574-5p treatment in VSMCs lowered contractile gene expression and increased secretory gene expression. These results suggest that miR-574-5p down-regulation induces VSMCs de-differentiation, consistently with miR-574-5p low expression observed in the aneurysmal aortic tissue. However, another study reported that miR-574-5p increased proliferation and decreased apoptosis of VSMCs from patients with coronary artery disease [31]. VSMCs receiving EVs from mice with aortic aneurysm displayed an increased expression of miR-574-5p and newly formed EVs. To our knowledge, production of EVs by VSMCs has not been documented in the aneurysmal context. However, in the vascular calcification process, VSMCs undergo phenotypic changes from a synthetic state toward a chondrogenic state, accompanied by a release of EVs [32]. All together, these data support a role for miR-574-5p in the aortic aneurysm formation, even though its implication in VSMCs proliferation remains unclear.

Mature miR-574-5p appears to be preferentially localized in the nucleus in VSMCs. This localization is unusual for a mature miRNA and this observation needs to be interpreted with caution considering the possibility of our FISH probe to recognize not only mature miR-574-5p but also precursor forms, as well as to the lack of appropriate control to exclude the detection of these precursors. Nevertheless, it suggests that this miRNA may play a regulatory role or regulate the expression of its target gene in the nucleus by a mechanism not yet elucidated. MiR-574-5p was found to be translocated from the nucleus towards cytoplasm and in vesicles newly formed that may be secreted as EVs after the Ang II treatment. This translocation appears to be associated with the decrease of miR-574-5p intracellular levels and an enrichment of miR-574-5p in EVs secreted by VSMCs. Interestingly, in human and murine aneurysmal context, a similar pattern of expression is observed, characterized by low intracellular levels of miR-574-5p in VSMCs and high levels of plasma levels of miR-574-5p, contained in EVs. MiR-574-5p down-regulation in the aortic tissue could be partly explained by a lowered transcription, since the pre-miR-574 is also down-regulated in the aneurysmal aortic tissue but in a lesser extent than mature miR-574-5p. Since Ang II is known to be involved in the TAA pathogenesis, is inducing a phenotypic switch of VSMC toward secretory state (by decreasing contractile gene expression, increasing the expression of secretory genes and inducing the EV formation), we postulate that the Ang II may be at least partly responsible for this specific pattern of miR-574-5p expression in the aneurysmal context.

MiR-574-5p was found to be expressed in plasma and EVs from *Fbn1^C1041G/+^* mice. This observation suggests that circulating miR-574-5p is, at least in part, transported in EVs in an aneurysmal context. This suggestion is strengthened by the ability of Ang II to induce EV production in VSMC. The presence of miR-574-5p in EVs documents an energy-dependent, selective and active secretion in the blood flow [21] by “donor” cells or tissue, toward targeted “recipient” cells or tissue. Several studies showed that miRNAs encapsulated in EVs can modify gene expression in target cells [33,34]. Especially, the VSMCs phenotype can be modulated by exosomal-miRNAs (such as miR-143/145 [34]) secreted by endothelial cells. These data support a “paracrine” role for miR-574-5p in TAA pathogenesis. Under stimulation of Ang II, VSMCs are able to create EVs containing miR-574-5p. Therefore, we can hypothesize that circulating miR-574-5p can originate from VSMC, and that Ang II may participate to its secretion but we have not provided evidence for its “recipient” cells or tissue.

Expression levels of miR-574-5p were inversely regulated between the aortic tissue and serum samples, a down-regulation being observed in aneurysmal aortic media compared to non-aneurysmal tissue, while serum levels of miR-574-5p were up-regulated in TAA patients. This expression pattern was also observed in *Fbn1^C1041G/+^* mice, a monogenic model that mimics the Marfan syndrome whereas patients had various and highly heterogenous etiologies, suggesting a high implication of miR-574-5p in TAA pathogenesis. According to the NCBI Gene database, the miR-574-5p sequence is localized in the *FAM114A1* gene and is highly conserved between human and mice and may suggest conservation of function. However, circulating levels of miR-574-5p were correlated with the aortic diameter of *Fbn1^C1041G/+^* mice, although they were not associated with the severity of the disease in patients. This discrepancy could be explained by the limited statistical power in human samples or by the heterogeneity of etiologies in patients while the *Fbn1^C1041G/+^* mouse model represents only one syndromic etiology. Indeed, the most prominent TAA aetiology in our two human cohorts is the bicuspid aortic valve, which shows common (*fbn1* gene expression decrease, immaturity of aortic wall) but also different histologic modifications compared to the Marfan syndrome [35]. Hence, the mouse model used in our study may not reproduce all TAA characteristics represented in our cohorts of patients.

In *Fbn1^C1041G/+^* mice, miR-574-5p appears to be modulated in several organs, and is highly up-regulated in kidneys. Kidneys are highly sensitive to blood pressure modulations and especially hypertension. It has been reported that patients with TAA were more likely to have renal disease than healthy people [36]. TAA causes hemodynamic modifications in circulating apparatus [37], that irrigates other organs. However, no differences of blood pressure have been observed between the Wt and *Fbn1^C1041G/+^* mice, therefore blood pressure alteration may not be associated with miR-574-5p up-regulation in kidneys. Even if kidney disease is not described in the Marfan syndrome and even if the renal structure is similar between the Wt and *Fbn1^C1041G/+^* mice, we cannot exclude that the alteration of miR-574-5p expression in the kidney is not a consequence of *fbn1* mutation. Interestingly, a case of a two-month old *Fbn1^C1041G/+^* mouse with unilateral renal systic disease has been recently reported, supporting a close monitoring of renal function in patients with the Marfan syndrome [38].

On the other hand, circulating levels of miR-574-5p were also up-regulated in patients with the coronary artery disease [39]. It would be conceivable that up-regulated circulating levels of miR-574-5p in TAA patients would originate from diseased coronary arteries. However, of the 28 TAA patients in our serum cohort, none had a reported coronary artery disease. Therefore, the up-regulation of circulating levels of miR-574-5p in TAA patients appears to be independent from the existing coronary artery disease and could be specific to the TAA diagnosis.

In future studies, miR-574-5p quantification may help improve risk stratification for the aortic dissection in TAA patients, presuming that the diagnostic value of miR-574-5p can be confirmed in independent and large patient cohorts. Further investigation of the origin and functional role of circulating miR-574-5p in the TAA pathogenesis may improve our understanding of this complex disease.

This study has several limitations. 1) The number of participants in the different cohorts and groups is low and TAA patients were younger than the controls in the serum cohort. 2) The association between the miR-574-5p and TAA was not validated in an independent and properly sized cohort. 3) Patients in this study present heterogenous TAA aetiologies. 4) The *Fbn1^C1041G/+^* mouse model may not represent this heterogeneity and our experimental results shall be extrapolated to human with caution. 5) A functional association between the miR-574-5p and TAA pathogenesis is strongly suggested by the bioinformatics analysis and its secretion in EVs, but this association should be further studied. 6) This study focused on one miRNA and the possibility that other miRNAs may also be associated with TAA cannot be excluded.

## 4. Materials and Methods

Microarray data have been made publicly available at the Gene Expression Omnibus under the accession number GSE110527 and can be accessed at https://www.ncbi.nlm.nih.gov/geo/query/acc.cgi.

Other data that support the findings of this study are available from the corresponding author upon reasonable request. Following a case-control design, we used two different cohorts of patients, a “tissue cohort” and a “serum cohort”. All supporting data are available in the Supplementary Materials.

### 4.1. Human Aortic Tissue Samples–“Tissue Cohort”

Aortic tissues samples were obtained from a patient cohort conducted at the Bichat Hospital in Paris, France, within the Cardiovascular Biobank, BRIF: BB-0033-00029 [40]. The clinical research protocol was approved by the local Ethics Committee (CPP 05 04 32, April 2005; updated in March 2008). Aneurysmal ascending aortic specimens were collected during aortic surgery which allowed a visual assessment of the presence of TAA in the ascending aorta, from 2006 onwards. Normal ascending aortas were obtained from deceased organ transplant donors with the authorization of the French Biomedicine Agency (PFS09-007) and in accordance with the Declaration of Helsinki, recovered from 2006 onwards. Deceased organ transplant donors were used as controls after verification of the absence of TAA by observation of the aorta during organ recovery. Aneurysmal tissues were sampled in the outer curvature, the most dilated part of the ascending aorta. Aortic tissue preparation consisted of an immediate dissection to separate medial and adventitial layers followed by freezing. This methodology allowed us to focus on VSMCs only present in the aortic media. Recovery of patient information was performed at the time of sampling or retrospectively from the hospital in charge of the patient. All protocols were performed in accordance with the principle of the Declaration of Helsinki and informed consents were obtained from all patients. Of this patient cohort, 20 patients with TAA were randomly selected, and were matched by age and sex with 20 healthy controls to be used in the present study.

### 4.2. Human Serum Samples–“Serum Cohort”

Blood samples were collected in dry tubes with gel from 32 TAA patients treated at the University Hospital of Liège from September 2009 to November 2015, and from 20 healthy individuals. The serum was separated by centrifugation for 10 min at 3000 g within 30 min after blood collection. The protocol was approved by the Ethics Committee of the University Hospital of Liège. For TTA patients, the TAA diagnosis was validated by the measurement of the aortic diameter at the aneurysm site using positron emission tomography–computed tomography. Controls did not have TAA as certified by echocardiography or scanner. Recovery of patient information was performed at the time of sampling or retrospectively from the hospital in charge of the patient. The participants gave written informed consent. Among the 32 TAA patients, two were excluded because of a TAA in the descending aorta, and two were excluded because of incomplete patient information.

### 4.3. Mice

All mice were cared for under strict compliance with the Partners Institutional Animal Care and Use Committee (IACUC), regulated by the United States Public Health Service (USPHS). Marfan (*Fbn1^C1041G/+^*) mice were acquired from Jackson laboratories (B6.129-Fbn1tm1Hcd/J, #01885).

For tissue analysis, animals were euthanized through inhalational isoflurane (Sigma, St, Louis, USA) prior to tissue collection. All tissue experiments were performed after sacrifice at six months. For ultrasounds, please see Supplementary Materials.

Dissected tissue was prepared for histology and staining as reported previously by Lino Cardenas et al. [41,42]. Peripheral blood was collected from Wt and *Fbn1^C1041G/+^* mice. Samples were centrifuged at 1500× *g* for 15 min to separate the plasma, and were frozen at –80°C until RNA extraction.

### 4.4. Isolation, Number and Size Distribution of Intact EVs

#### 4.4.1. Isolation of Intact EVs

We pooled the collected plasma or conditioned medium from VSMCs from each group and used the cushioned-density gradient ultracentrifugation method as described by Li et al. [43]. In brief, ~1 mL of plasma from each group was diluted with PBS (phosphate-buffered saline) to a total volume of 8 mL, placed over 2 mL of 60% iodixanol solution (SKU:D1556, Millipore Sigma, Bedford, USA) and centrifuged at 110,000× *g* (k factor of 124) for 2 h at 4 °C using an SW41Ti swinging-bucket rotor (Beckman Coulter, Danvers, USA). Following this step, we carefully aspirated and discarded the top 7 mL and recovered the bottom 3 mL, which contained the EVs fraction. For the second density gradient ultracentrifugation step, we transferred the 3 mL in a new tube, mixed well, carefully overlaid three layers of 20%, 10%, and 5% iodixanol (3 mL each), and centrifuged at 110,000× *g* for 18 h at 4 °C. We then collected 12 fractions (1 mL each) from the top to the bottom of each tube and pooled fractions 6–10 that are enriched for EVs for downstream experiments. To remove iodixanol from the recovered EVs, we performed a dialysis step in a filtered (0.22 μm) 1× PBS buffer using a 100 kD Float-A-Lyzer G2 Dialysis device (SKU:G235059, Spectrum Lab, Rancho Dominguez, CA) according to the manufacturer’s protocol.

#### 4.4.2. Number and Size Distribution of EVs

To measure the concentration and size distribution of isolated EVs we used a NanoSight LM10 system (Malvern Panalytical, Malvern, UK). In brief, we diluted EV samples 1:20 with filtered (0.22 μm) 1× PBS to achieve the instrument’s optimal particles per frame range. At the end of each measurement, the pump was washed several times with filtered 1× PBS to avoid cross-sample contamination. The camera settings were adjusted per the manufacturer’s instructions, samples were recorded in three repeats of 20 s, and data were analyzed using the NanoSight NTA 2.3 software (Malvern Panalytical, Westborough, USA).

### 4.5. Primary VSMCs Isolation and Culture

Mouse aortic VSMCs were isolated by the standard explant of the ascending section of the aortas as previously described [42]. Human aortic VSMCs for a healthy donor were purchased from Cell Application Inc., San Diego, USA (354K-05a). The VSMC identity was assessed by immunofluorescence staining of contractile markers, including SM22α, calponin-1, smoothelin, and vinculin. In order to preserve cell identity, all experiments were carried out at passages 1–5. VSMCs were grown with the smooth muscle cells growth medium from Cell Applications Inc., (catalog 311-500). For the Ang II treatment, primary human VSMCs were treated or not with 1 µM of Ang II for 24 h and fixed before sequential FISH.

### 4.6. RNA Extraction

For human aortic tissues samples, RNA extraction was performed with the mirVana isolation kit (Life Technologies, Carlsbad, USA). The MiRNeasy serum/plasma kit (Qiagen, Hilden, Germany) was used for human serum and murine plasma samples, and for VSMCs, mice aortic and EVs samples, RNA extraction was done using the miRNeasy kit (Qiagen). See Supplementary Materials for further information.

### 4.7. Microarrays

Agilent G3 human miRNA microarrays containing 5893 probes corresponding to 2549 miRNAs from miRBase release 21 were used, with the miRNA complete labeling and hybridization kit (Agilent, Santa Clara, USA), according to the manufacturer’ recommendations [44,45]. See Supplementary Materials for further information.

### 4.8. Bioinformatics Analyses

To identify genes known to be associated with the TAA pathogenesis, we used the following keywords in the NCBI gene database: (Thoracic AND aortic AND aneurysm) OR (Destructive AND “extracellular matrix” AND remodeling AND aorta) OR (Elastin AND fragmentation AND aorta AND media) OR ((“Vascular smooth muscle cells” OR “Vascular smooth muscle cell”) AND (proliferation OR secretion OR migration OR apoptosis)) OR (“Extracellular matrix” AND (homeostasis OR structure)) OR (Inflammation AND “extracellular matrix” AND aorta AND media) OR (Elastogenesis AND aorta AND media) OR (Aorta AND vasculogenesis). The following 12 genes known to be involved in monogenic TAA or dissection were also selected: *FBN1*; *EFEMP2*; *COL3A1*; *TGFBR1*; *TGFBR2*; *SLC2A10*; *NOTCH1*; *JAG1*; *SMAD3*; *ACTA2*; *MYH11*; *FLNA* [15]. Predicted interactions between differentially expressed miRNAs and TAA-associated genes were obtained using TargetScan [46], miRDB [47] and DIANA microT-CDS [48]. Protein-protein interactions between TAA-associated genes were obtained from the IntAct database [49]. The interaction network of miRNAs and genes was generated using Polar Mapper [50]. Enriched biological processes and pathways were analyzed with DAVID, the Database for Annotation, Visualization and Integrated Discovery [51].

### 4.9. Reverse Transcription and Quantitative PCR

Human aortic tissues and serum samples, as well as murine plasma samples were reverse-transcribed using the miScript II reverse transcription kit (Qiagen) according to the provider instructions. PCR were run on the CFX96 thermocycler (Bio-Rad, Hercules, USA).

For mice aortic tissues and EVs, the miRNA cDNA synthesis was prepared using the TaqMan Advanced miRNA cDNA Synthesis Kit (Thermofisher scientific, Waltham, USA) following the manufacturer’s protocol. See Supplementary Materials for further information.

### 4.10. Sequential FISH and Immunofluorescence Microscopy

miRCURY-FISH Probe recognizing murine miR-574-5p (YD00616643-BED) labeled with 5’-FAM and 3’-FAM (Qiagen) was hybridized to tissue samples at 65 °C for 4 h, followed by incubation with the F-actin conjugated antibody (ActinGreen™ 488 ReadyProbes, ThermoFisher Scientific), and with CD63, CD9 or Fetuin antibodies. Two-dimensional and white light images were analyzed and quantified using the ImageJ software (version 1.8.0_112, National Institutes of Health, Bethesda, USA).

### 4.11. Statistical Analyses

Results are presented as a median ± standard deviation. SigmaPlot (version 12.5, Systat Software Inc., San Jose, CA, USA) was used to perform the statistical analyses. The Shapiro-Wilk normality test preceded all analyses. The *T*-test and Mann–Whitney test were used to compare two groups of continuous variables following Gaussian and non-Gaussian distributions, respectively. The Chi-square and Fisher Exact test were used for qualitative variables. Correlations between two variables were assessed using the Spearman test. The association between miR-574-5p and TAA in the serum cohort was evaluated using the ROC (receiver operating characteristic) curve analysis.

## 5. Conclusions

We identified an association between miR-574-5p and TAA and we propose that these miRNAs may be considered as a potential biomarker to diagnose TAA. We also propose that miR-574-5p may act as a paracrine mediator in the TAA pathogenesis and could be secreted in answer to Ang II. Further studies are warranted to confirm these concepts.

## Supplementary Materials

Supplementary Materials, Supplementary Figures S1–S6 and Supplementary Tables S1–S7 can be found in the Supplementary Materials file at https://www.mdpi.com/1422-0067/20/16/3924/s1.

## Abbreviations

Ang II	Angiotensin II
EV	Extracellular vesicle
Fbn1	Fibrillin 1
FISH	Fluorescence in situ hybridization
MiRDB	microRNA Target Prediction Database
MiRNA	MicroRNA
NCBI	National Center for Biotechnology information
TAA	Thoracic aortic aneurysm
VSMC	Vascular smooth muscle cell
Wt	Wild type
