# Supplementary material for: MiR-574-5p: A Circulating Marker of Thoracic Aortic Aneurysm
Source: Int J Mol Sci. 2019 Aug 12;20(16):3924. doi: 10.3390/ijms20163924 (PMC6720007; doi:10.3390/ijms20163924)
Supplement: Supplementary file 1 [file ijms-20-03924-s001.zip › ijms-552299-supplementary/Supplementary material Int J Mol Sci.pdf]

SUPPLEMENTAL MATERIAL

Supplementary materials

1. Mice ultrasounds

For ultrasounds on mice, a Nair hair removal cream was used on all mice the day prior to ultrasounds. All ultrasounds were performed on awake, unsedated mice using the Visualsonics Vevo660 imaging system and a 30 MHz transducer. The aorta was imaged using a standard parasternal long axis view. Dimensions from each animal represent averages of measurements made on still frames in systole of the maximal internal diameter of the aortic valve annulus, aortic sinuses, sinotubular junction, or ascending aorta by a cardiologist blinded to genotype. On the basis of historic controls, the mean diameter of the Wt (wild-type) aorta at 6 months of life is 1.75 + /-0.2 mm while in our disease model ($Fbn1^{C1041G/+}$) the mean diameter is 2.2 ± 0.3 mm. We calculated a need for ~10 animals per group at a power (1-β) of 0.8 and a Type I error rate (α) of 5% to detect a 0.34 mm (57%) mean improvement (vs. 2.2mm mean) improvement in aneurysm diameter. The ultrasound resolution is between 30 and 100 microns with 5-15 mm penetration.

2. RNA extraction procedure.

For human aortic tissues samples, approximatively 45 mg of aortic media tissue was crushed in lysis binding buffer (mirVana isolation kit, Life Technologies, Carlsbad, USA). Total RNA was then extracted using miRVana kit (Life Technologies), according to provider recommendations. Briefly, a small volume of miRNA homogenate additive (1/10 of the lysate volume) was added to the lysate, followed by the addition of one volume of acid phenol:chloroform. After a centrifugation step, the aqueous phase was recovered and washed through a column using washing solutions containing increasing ethanol concentration. Total RNA was eluted from the column with 50µl of RNase-free DNase-free water. RNA concentration was measured using a Nanodrop spectrophotometer.

For human serum samples, RNA was extracted from 90µl of serum, added with 110µl of molecular grade phosphate buffered saline using miRNeasy serum/plasma kit (Qiagen, Hilden, Germany) and the semi-automated QIAcube system, according to manufacturer recommendations. Briefly, samples were homogenized with Qiazol (Qiagen), and 1.6×10^8 copies of spike-in cel-miR-39 were added in the samples, followed by the addition of 200µl of chloroform and a centrifugation step. The aqueous phase was recovered and introduced into the QIAcube apparatus for several washes using a column-based system and solutions containing increasing ethanol concentrations. Total RNA was finally eluted in 18µl of water.

For VSMCs, mice aortic and EVs samples, total RNA was prepared from the ascending aortas or EV-fractions 6-10 using miRNeasy kit (Qiagen) following the manufacturer's protocol.

For murine plasma samples, RNA was extracted from 50µl of serum using miRNeasy serum/plasma kit (Qiagen) according to manufacturer recommendations. Briefly, samples were homogenized with Qiazol (Qiagen), and 1.6×10^8 copies of spike-in cel-miR-39 were added in the samples, followed by the addition of 200µl of chloroform and a centrifugation step. The aqueous phase was recovered and passed through a silica membrane. The membrane was washed several times with solutions containing increasing concentrations of ethanol. Total RNA was finally eluted in 18µl of water.

3. Microarrays

For each tissue sample, 100ng of RNA was successively dephosphorylated, denatured, ligated with cyanine3 fluorophore, dried and hybridized onto microarrays overnight at 55°C. Microarray

slides were washed and scanned using the high-resolution scanner G2565C (Agilent). Quantifiable values of microarray were extracted using Agilent's feature extraction software. Of the 40 samples, 2 paired-samples were excluded because of poor quality. MiRNA expression data were computed and normalized using the Robust Microarray Average method integrated in AgiMicroRna R package. The data has been deposited in the GEO repository under accession number GSE110527. Differentially expressed miRNAs were obtained after fitting a linear model and applying Empirical Bayes method from Limma R package. Heatmap was generated using Clucter3 [1] and Java TreeView [2].

4. Reverse transcription and quantitative PCR

450 to 500 ng of total RNA from human aortic tissues samples were reverse-transcribed using miScript II reverse transcription kit (Qiagen) with the HiFlex buffer. For measurement of miRNA expression, the miScript SYBR Green PCR (Qiagen) system was used. SNORD68 was used as housekeeping gene for normalization of quantitative PCR raw data in aortic tissue samples. For measurement of pre-miR-574, the miRCURY LNA PCR system (Qiagen) was used. Expression levels of pre-miR-574 were calculated using the formula $2^{\text{mean Cq all samples - Cq sample}}$. For human serum and murine plasma samples, 12µl of total RNA were reverse-transcribed using miScript II reverse transcription kit (Qiagen) with the HiSpec buffer and cel-miR-39 was used for normalization. MiR-574-5p primers were obtained from Qiagen. PCR were run on the CFX96 thermocycler (Bio-Rad) with the miScript SYBR Green PCR system (Qiagen).

For mice aortic tissues and EVs, U6 was used as housekeeping gene for normalization in aortic tissue samples. For EVs, different miRNAs were tested by quantitative PCR for their stability across samples (miR-133a-3p, miR-133a-5p, miR-199a-5p, miR-191-5p, miR-29-3p) and miR-191-5p was selected for normalization.

For VSMCs, the High-Capacity RNA-to-cDNA kit was used, according to manufacturer's protocol. GAPDH was used as housekeeping gene for normalization. For primer sequence or reference, please see the Major Resources Table in the Supplemental Material.

Supplementary figures

Figure S1: Study design.

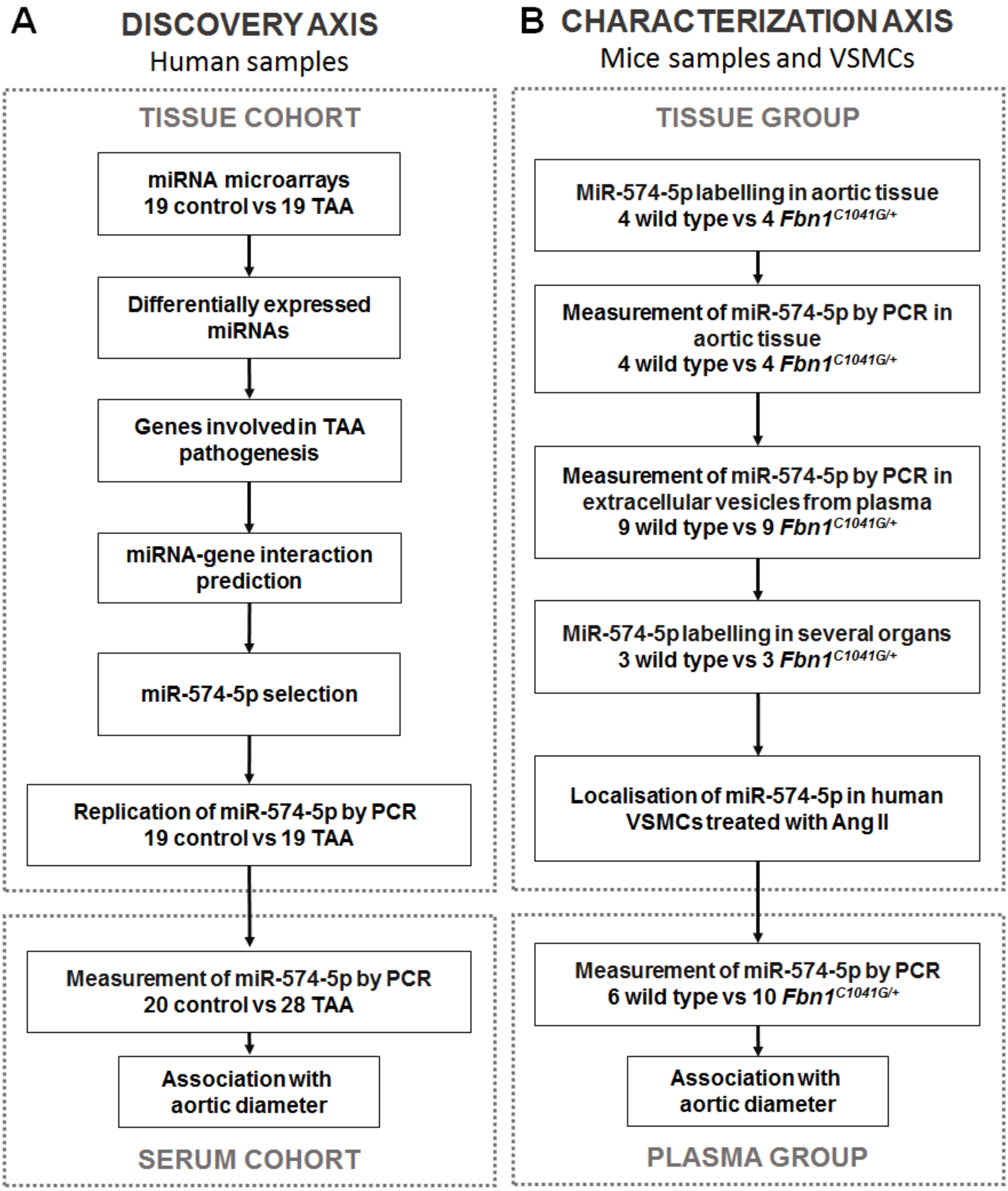

Figure S2: MiRNA-gene and protein-protein interaction network: polar map of the interaction network. Eight modules representing high density of links between miRNAs and genes are displayed.

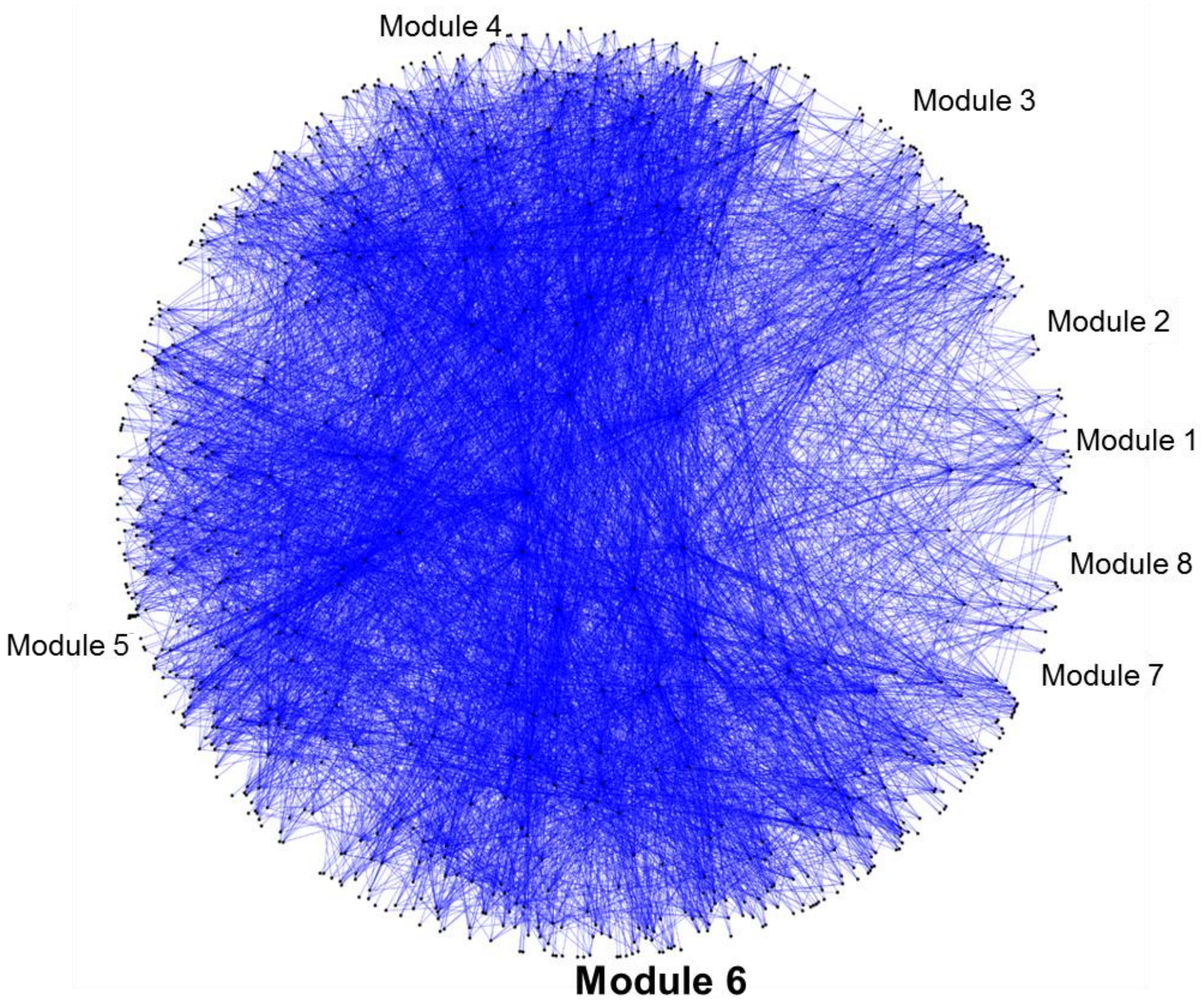

Figure S3: **miR-574-5p expression in tissue (A) and serum (B) cohort according to the etiology of the aneurysm.** Tissue cohort: dissection n=2; Marfan syndrome n=2; Bicuspid aortic valve n=9; Degenerative etiology n=2; unknown n=4 and control n=19. Serum cohort: dissection n=3; Bicuspid aortic valve n=6; Ehler-Danlos syndrome n=1; unknown n=18 and control n=20. *p<0.05; **p<0.01 and ***p<0.001.

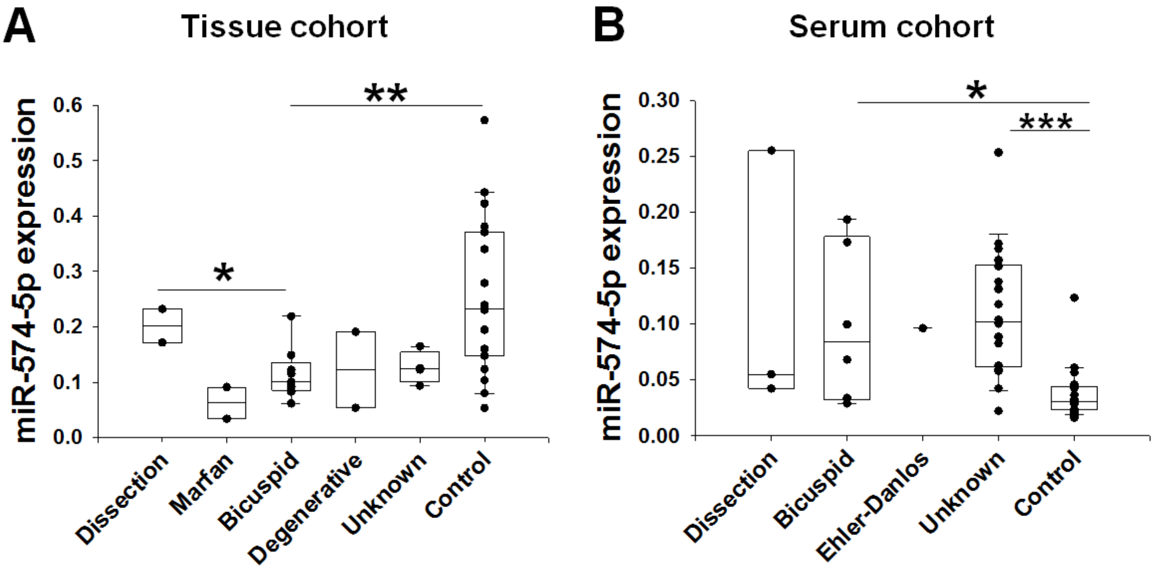

Figure S4: **miR-574-5p expression in serum cohort according to hyperlipidemia (A) and tobacco (B) status.** For control group: 4 patients had hyperlipidemia and 16 had not; tobacco status: never n=14; stopped n=6. For TAA group, 15 patients had hyperlipidemia and 13 had not; tobacco status: never n=11; stopped n=8; current use n=8; unknown n=1. *p<0.05; **p<0.01 and ***p<0.001.

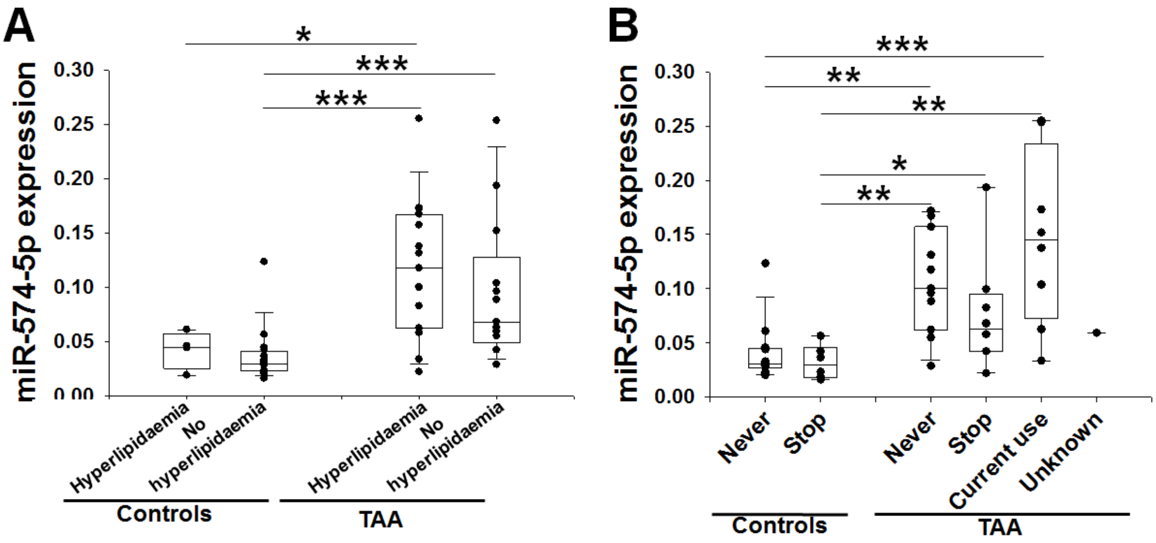

Figure S5: Localization of miR-574-5p in primary vascular smooth cells (VSMCs) from wild-type (Wt) mice, treated with plasma extracellular vesicles (EVs) from Wt and *Fbn1*$^{C1041G/+}$ mice. Immunofluorescence and FISH staining of miR-574-5p (red); CD63, a membrane marker of EVs (green) and nucleus (blue) were used on primary VSMCs. The yellow scale bar corresponds to 500µm. Cells were isolated from 3 male mice.

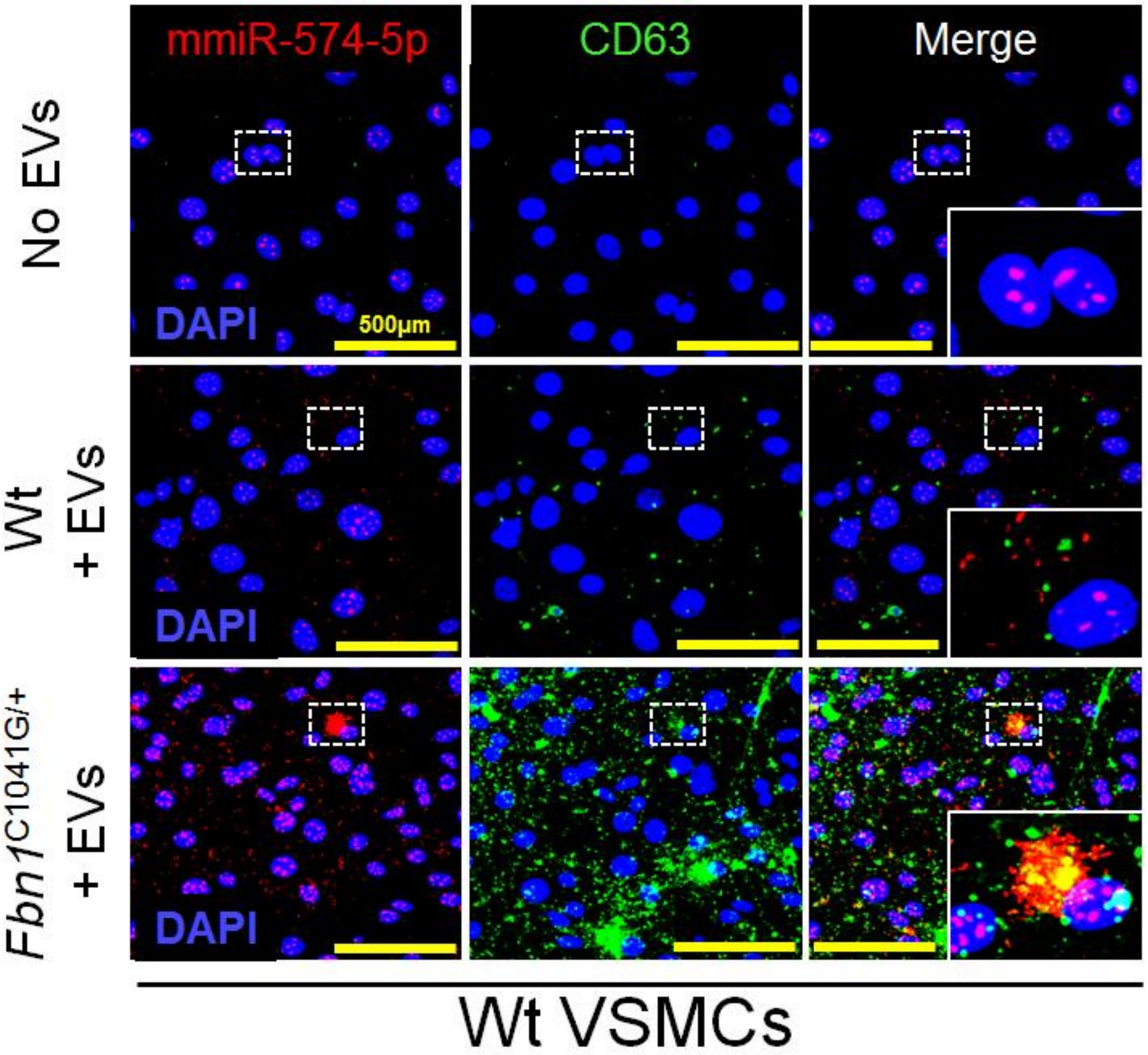

Figure S6: Characterization of aortic feature in *Fbn1*$^{C1041G/+}$mice model : diameter of different part of the aorta, measured by echography; respectively, aortic valve diameter **(A)** , aortic root diameter **(B)** , sino-tubular junction diameter **(C)** , ascendant aorta diameter **(D)** and weight **(E)**. *p < 0.05.

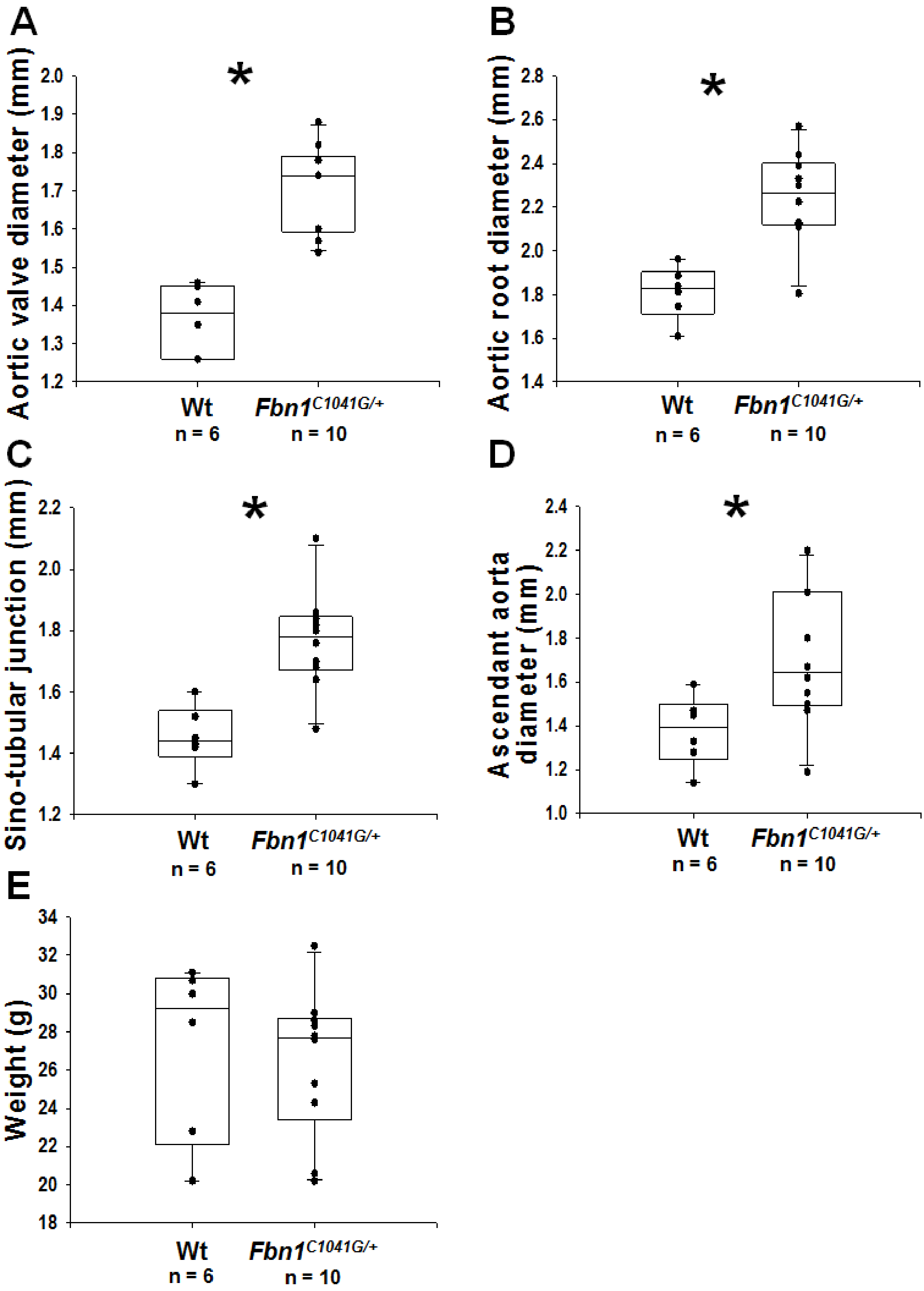

Supplementary tables

Table S1. Patient characteristics (tissue cohort).

	Control (n=19)	TAA (n=19)	p-value (Control vs. TAA)
**Age, median (range)**	56 (36-76)	57 (35-77)	0.96
**Sex, male, n (%)**	13 (74)	13 (74)	0.727
**Aortic diameter in mm, median (range)**	N/A	52 (33-64)	
**Aortic dissection, n (%)**	N/A	2 (10.5)	
**Aortic aneurysm aetiology, n (%)**			
Marfan	N/A	2 (10.5)	
Degenerative	N/A	2 (10.5)	
Bicuspid aortic valve	N/A	9 (47.4)	
Ehler-Danlos (type IV)	N/A	0 (0)	
Unknown	N/A	4 (21.1)	
**Smoking status**			
Never	N/A	1 (5.3)	
Stop	N/A	3 (15.8)	
Current	N/A	5 (26.3)	
Unknown	N/A	10 (52.6)	
**Medication**			
Aspirin	N/A	1 (5.3)	
Angiotensin II receptor blocker	N/A	3 (15.8)	
Statin	N/A	3 (15.8)	
Beta-blockers	N/A	4 (21.1)	
Inhibitors of conversion enzyme	N/A	2 (10.5)	
Unknown	N/A	11 (58)	

TAA : thoracic aortic aneurysm; N/A: not available.

Table S2. Patient characteristics (serum cohort).

	**Control (n=20)**	**TAA (n=28)**	**p-value (Control vs. TAA)**
**Age, median (range)**	75 (67-85)	61 (32-84)	<0.001
**Sex, male, n (%)**	16 (80)	21 (75)	0.741
**Aortic diameter in mm, median (range)**	N/A	48 (35-74)	
**Aortic dissection, n (%)**	N/A	3 (10.7)	
**Aortic aneurysm aetiology, n (%)**			
Marfan	N/A	0 (0)	
Degenerative	N/A	0 (0)	
Bicuspid aortic valve	N/A	6 (21.4)	
Ehler-Danlos (type IV)	N/A	1 (3.6)	
Unknown	N/A	18 (64.3)	
**Hyperlipidemia, n (%)**	4 (20)	15 (53.6)	0.041
**Smoking status**			0.036
Never	14 (70)	11 (39.3)	
Stop	6 (30)	8 (28.6)	
Current	0 (0)	8 (28.6)	
Unknown	0 (0)	1 (3.5)	
**Medication**			
Aspirin	9 (45)	13 (46.4)	0.845
Clopidogrel	1 (5)	1 (3.6)	0.625
Statin	9 (45)	15 (53.6)	0.770
Beta-blockers	9 (45)	13 (46.4)	0.226
Angiotensin-converting enzyme inhibitors	4 (20)	2 (7.1)	0.218
None	4 (20)	5 (17.9)	0.851

TAA : thoracic aortic aneurysm; N/A: not available.

Table S3 – Patient characteristics (tissue cohort compared with serum cohort)

	**Tissue cohort (n=38)**	**Serum cohort (n=48)**	**p-value**
**Age, median (range)**	56 (36-77)	71 (32-85)	<0.001
**Sex, male, n (%)**	26 (68%)	37 (77%)	0.512
**Aortic diameter in mm, median (range)**	52 (33-64)	48 (35-74)	0.431
**Aortic dissection, n (%)**	2 (5%)	3 (6%)	0.787
**Aortic aneurysm aetiology, n (%)**			0.243
Marfan	2 (5%)	0	
Degenerative	2 (5%)	0	
Bicuspid aortic valve	9 (24%)	6 (12%)	
Ehler-Danlos (type IV)	0	1 (2%)	
**Smoking status**			<0.001
Never	1 (3%)	25 (52%)	
Stop	3 (8%)	14 (29%)	
Current	5 (13%)	8 (17%)	
Unknown	29 (76%)	1 (2%)	
**Medication**			
Aspirin	1 (3%)	22 (46%)	<0.001
Statin	3 (8%)	24 (50%)	<0.001
Beta-blockers	4 (10%)	22 (46%)	<0.001
Inhibitors of conversion enzyme	2 (5%)	6 (12%)	0.293
None	0 (0%)	9 (19%)	0.014
Unknown	30 (79%)	0	<0.001

Missing data: in tissue cohort: for aortic diameter, n=4; for smoking status, n=29; for medication, n=30.

Table S4. List of 690 TAA-associated genes identified using NCBI Gene database and literature

A2M	CCNE1	EGLN1	IGF1	MIR15A	PKN1	SPARC
AAT1	CCNE2	EIF2AK2	IGF1R	MIR21	PLA2G2A	SPARCL1
AAT2	CCR6	ELANE	IGFBP5	MIR210	PLAT	SPINT1
ABCA1	CCR8	ELAVL1	IL10	MIR223	PLAU	SPON1
ABL1	CD151	ELN	IL17A	MIR24-1	PLAUR	SPON2
ABL2	CD209	EMILIN1	IL17F	MIR26A1	PLCG1	SPP1
ACAN	CD248	EMILIN2	IL18	MIR29A	PLEC	SPRR3
ACE	CD34	ENAM	IL19	MIR31	PLG	SPRY2
ACE2	CD4	ENG	IL1A	MIR638	PLIN2	SRC
ACKR3	CD40	ENO1	IL1B	MIR663A	PLOD2	SREBF1
ACTA2	CD40LG	ENPP1	IL1RL1	MIRLET7G	PODN	SRF
ACTN1	CD44	EPC1	IL22	MMP1	POLDIP2	SRI
ADAM10	CD47	EPHA2	IL6	MMP10	POMT1	SSPN
ADAM15	CDC42	ERG	ILK	MMP12	POSTN	ST14
ADAM17	CDH1	ERO1A	INHBA	MMP13	POU1F1	ST6GALNAC6
ADAM8	CDH2	ERO1B	IRS1	MMP14	PPARA	ST8SIA1
ADAMTS10	CDK6	ETS1	ITGA1	MMP16	PPARD	STAT1
ADAMTS13	CDKN1A	EZR	ITGA2B	MMP2	PPARG	STIM1
ADAMTS14	CDKN1B	F11R	ITGA5	MMP28	PPARGC1A	STK11
ADAMTS20	CEL	F2	ITGA8	MMP3	PPIB	TBK1
ADAMTS4	CFHR1	F2R	ITGAL	MMP7	PRDX4	TEK
ADAMTS5	CHAD	F2RL1	ITGAM	MMP8	PREB	TFF3
ADCYAP1	CHI3L1	F2RL3	ITGAV	MMP9	PRF1	TFPI2
ADGRG1	CHN2	F3	ITGAX	MRC2	PRKCA	TGFB1
ADIPOQ	CHST3	FASLG	ITGB1	MSN	PRKCB	TGFB2
ADM	CIAPIN1	FBLN5	ITGB2	MSRA	PRKCD	TGFB3
ADORA2B	CKLF	FBN1	ITGB3	MSTN	PRKCE	TGFBI
ADRA1A	CLCN3	FBN3	ITGB4	MTHFR	PRKCZ	TGFBR1
ADRA1B	CLEC11A	FER	ITGB5	MTOR	PRKD1	TGFBR2
ADRA2A	CLIC4	FGA	ITGB6	MUC17	PRKG1	TGM2
ADRA2B	CLU	FGB	ITLN1	MUC4	PRSS3	THBD
ADRA2C	CMA1	FGF1	JAG1	MUC5AC	PSAP	THBS1
AEBP1	COCH	FGF18	JAM3	MYB	PSEN1	THBS4
AGER	COL10A1	FGF2	JUN	MYC	PTEN	THY1
AGT	COL13A1	FGF20	KCNA10	MYCN	PTGFR	TIMP1

AGTR1	COL14A1	FGF23	KCNA3	MYH11	PTGIS	TIMP2
AGTR2	COL15A1	FGF9	KCNMA1	MYH9	PTGS2	TIMP3
AIF1	COL17A1	FGFR2	KDR	MYLK	PTH	TJP2
AKT1	COL18A1	FGFR4	KIT	MYOC	PTK2	TLN1
AKT2	COL1A1	FGFRL1	KITLG	MYOCD	PTK2B	TLR2
ALOX12	COL1A2	FGG	KLF4	NCL	PTPRZ1	TLR4
ALOX15	COL25A1	FHOD1	KLF6	NCOA3	PTX3	TLR9
ALOX15B	COL28A1	FKBP1A	KLK2	NCSTN	PVR	TM4SF5
ALOX5	COL2A1	FLG	KLK4	NDP	QSOX1	TMPRSS 6
ALPL	COL3A1	FLNA	KLK7	NDRG2	RAB25	TNF
AMD1	COL4A1	FLT1	KLKB1	NDRG4	RAC1	TNFAIP6
AMELX	COL4A2	FMOD	KRT1	NDUFS3	RCAN1	TNFRSF1 1B
ANGPT1	COL4A3	FN1	LAMA1	NEDD4	RDX	TNFRSF1 A
ANGPTL4	COL6A3	FOXF1	LAMA2	NF1	RELN	TNFRSF1 B
ANTXR1	COL7A1	FOXF2	LAMA4	NFAT5	RETN	TNFSF11
ANTXR2	COL9A1	FRAS1	LAMA5	NFATC1	RHEB	TNFSF12
ANXA2	COMP	FSCN1	LAMB1	NFKB1	RHOA	TNK2
APCS	CPB2	FSTL1	LAMB2	NFKB2	ROCK1	TNNI2
APLN	CREBBP	FURIN	LAMC1	NID2	ROCK2	TNXB
APLP2	CREG1	FYN	LAMC2	NOD2	RPSA	TP53
APOA1	CRISP3	GAS6	LAMC3	NOS2	RPTOR	TP63
APOD	CRP	GATA6	LAMP1	NOS3	RTN4	TP73
APOE	CRTAC1	GC	LARGE	NOTCH1	RUNX2	TPSAB1
APOH	CSF2	GDF5	LATS2	NOTCH2	S100A12	TRAPPC4
APP	CSGALN ACT1	GNB2L1	LEP	NOTCH3	S100A4	TRIB1
AQP1	CST3	GPC1	LEPR	NOX1	SDC1	TRPC1
AR	CTGF	GPC3	LGALS1	NOX4	SDC2	TRPC6
ARF6	CTNNB1	GPC4	LGALS3	NPM1	SDC4	TRPM7
ARHGAP 9	CTSB	GPER1	LGALS8	NPPA	SEL1L	TTN
ARHGEF 25	CTSD	GREM1	LGALS9	NPPB	SERAC1	TTR
ARNTL	CTSK	GSK3B	LIMK1	NPY2R	SERPINA 1	TWIST1
ATP2A2	CTSL	GSN	LMNA	NQO1	SERPINB 5	TYK2
ATP7A	CTSS	GSR	LOX	NR1H2	SERPINB 9	TYMP
AURKA	CTTN	GZMB	LOXL1	NR1H3	SERPINE 1	UCHL1
AXL	CX3CL1	HAS1	LPL	NR1I2	SERPINE	UGDH

					2	
BAD	CX3CR1	HBEGF	LRP1	NR3C2	SERPINF<br>1	UPF1
BAG1	CXCL10	HES1	LRP5	NR4A3	SFRP1	USF2
BAX	CXCL12	HEXIM1	LRP6	NRG1	SFTPA1	VASP
BCL2	CXCL8	HEY1	LTB4R	NRP1	SFTPA2	VCAM1
BGN	CXCR4	HEY2	LTBP1	NRXN1	SFTPD	VEGFA
BIRC2	CYBA	HGF	LTBP2	NTM	SHH	VEGFB
BIRC5	CYR61	HIF1A	LTF	OLFML2A	SIRPA	VTN
BMP1	CYSLTR1	HMGB1	LYZ	OLR1	SIRT1	VWA1
BMP2	DAG1	HMGCR	MADCAM<br>1	ORAI1	SLC20A1	VWF
BMP4	DCN	HMOX1	MAP3K1	OSM	SLC2A10	WISP1
BMP7	DDR1	HNRNPA<br>2B1	MAPK1	P4HB	SLC2A9	WISP3
BRCA1	DDR2	HOXA10	MAPK14	PAK1	SLIT2	WNT1
BSG	DDX39B	HPSE	MAPK3	PAK2	SMAD2	WNT3A
BST1	DDX58	HRAS	MATN2	PCNA	SMAD3	WNT5A
C5	DEFA1	HSP90AA<br>1	MATN3	PCOLCE	SMAD4	WNT7B
CALR	DMBT1	HSP90B1	MCAM	PCSK9	SMAD7	WTAP
CAMP	DMD	HSPA5	MDM2	PDGFA	SMPD2	WWOX
CAPN1	DMP1	HSPB1	MEF2C	PDGFB	SNAI1	YAP1
CAPN2	DPP4	HSPG2	MEP1B	PDGFRB	SNAP23	YWHAG
CAPNS1	DST	HTRA1	MEPE	PEA15	SNX17	ZNF410
CASK	E2F1	HTT	MESP1	PEBP1	SOCS1	
CASP3	ECM1	HYAL1	MET	PECAM1	SOD1	
CASR	EDIL3	HYAL2	MFAP5	PGF	SOD2	
CAST	EDN1	HYAL4	MFGE8	PHEX	SOD3	
CAT	EDNRA	ICAM1	MFI2	PI3	SORL1	
CAV1	EDNRB	ICAM2	MFN2	PIK3CB	SOST	
CCL2	EFEMP1	ID1	MGP	PIK3CG	SOX9	
CCL20	EFEMP2	IDO1	MIA	PIN1	SP1	
CCL7	EGFL7	IER3	MIR143	PITX2	SP7	
CCND1	EGFR	IFNG	MIR145	PKM	SPACA3	

Table S5 – Biological processes enriched in module 1; module 3; module 4; module 5; module 6 and module 7. FDR : false discovery rate – Please see Appendix B.

Table S6 –MiRNAs included in the module 6

miRNA	Traffic	Fold change (log2)	Expression mean (control and TAA)	Adjusted p-value
hsa-miR-106b-5p	1717.16	0.83	8.54	1.97E-03
hsa-miR-1180-3p	502.00	-1.89	5.19	4.87E-10
hsa-miR-125a-5p	2016.02	0.88	8.75	4.55E-03
hsa-miR-125b-5p	808.85	0.77	13.45	3.10E-04
hsa-miR-127-3p	550.74	1.74	6.46	7.12E-06
hsa-miR-128-3p	6186.65	1.75	7.24	4.40E-05
hsa-miR-132-3p	1546.18	0.88	5.34	3.48E-03
hsa-miR-1-3p	1226.55	0.66	8.78	3.04E-02
hsa-miR-140-5p	1850.69	0.84	11.91	2.72E-04
hsa-miR-149-5p	7160.61	0.86	5.15	1.02E-04
hsa-miR-151a-5p	809.71	1.02	8.9	5.88E-04
hsa-miR-15a-5p	2117.39	0.68	9.66	1.03E-02
hsa-miR-1915-3p	948.96	-0.51	11.47	4.88E-03
hsa-miR-193a-5p	3553.22	0.93	6.86	3.24E-04
hsa-miR-193b-3p	502.00	0.92	8.88	1.30E-04
hsa-miR-20b-5p	1535.46	0.94	7.24	2.52E-03
hsa-miR-214-3p	3353.07	0.97	9.61	6.49E-05
hsa-miR-23a-3p	3418.89	0.57	13.78	8.41E-04
hsa-miR-24-3p	6353.67	0.55	13.29	1.01E-03
hsa-miR-28-5p	1136.07	0.95	7.87	2.83E-03
hsa-miR-2861	538.64	-0.58	11.73	1.07E-02
hsa-miR-3127-5p	584.93	-2.09	6.09	6.39E-08
hsa-miR-324-3p	737.89	0.62	9.47	2.83E-03
hsa-miR-324-	1307.46	1.25	7.41	1.84E-05

5p				
hsa-miR-331-3p	4821.11	1.07	8.86	5.27E-04
hsa-miR-342-3p	5387.52	1.14	8.73	4.88E-05
hsa-miR-34a-5p	3640.53	0.58	11.05	9.10E-03
hsa-miR-365a-3p	2441.28	0.79	10.08	1.10E-03
hsa-miR-3665	502.96	-0.55	10.78	3.66E-03
hsa-miR-377-3p	3112.98	1.88	5.62	8.72E-07
hsa-miR-423-5p	2606.54	0.56	7.59	3.20E-03
hsa-miR-4261	595.33	1.29	6.05	3.77E-04
hsa-miR-4281	1002.00	-0.97	12.41	5.95E-03
hsa-miR-4306	515.86	0.65	7.12	1.69E-04
hsa-miR-4324	539.02	0.89	5.98	1.52E-05
hsa-miR-4459	820.04	-0.67	11.49	9.38E-03
hsa-miR-4515	524.97	0.51	6.42	7.08E-03
hsa-miR-4669	505.03	-0.69	8.29	8.69E-04
hsa-miR-4726-5p	502.00	-3.42	5.95	2.21E-10
hsa-miR-4728-5p	1756.83	0.7	8.82	0.042817789
hsa-miR-4770	502.00	1.07	5.92	5.72E-04
hsa-miR-484	2470.29	1.04	6.07	3.57E-05
hsa-miR-487b-3p	550.67	1.62	5.98	3.75E-05
hsa-miR-564	1365.46	0.6	6.69	2.95E-04
hsa-miR-574-5p	4288.79	-1.2	8.87	2.79E-06
hsa-miR-638	556.09	-0.54	12.02	1.27E-02
hsa-miR-670-5p	663.53	-3.42	5.05	5.31E-11
hsa-miR-874-3p	3827.13	0.75	7.19	1.48E-05
hsa-miR-93-5p	1162.43	0.84	7.74	2.79E-03

Table S7 -Top-10 traffic miRNAs from module 6.

miRNA	Traffic	Fold change (log2)	Expression mean (control and TAA)	Adjusted p-value
**miR-574-5p**	**4288.79**	**-1.20**	**8.87**	**2.79E-06**
miR-874-3p	3827.13	0.75	7.19	1.48E-05
miR-128-3p	6186.65	1.75	7.24	4.40E-05
miR-342-3p	5387.52	1.14	8.73	4.88E-05
miR-149-5p	7160.61	0.86	5.15	1.02E-04
miR-193a-5p	3553.22	0.93	6.86	3.24E-04
miR-331-3p	4821.11	1.07	8.86	5.27E-04
miR-23a-3p	3418.90	0.57	13.78	8.41E-04
miR-24-3p	6353.67	0.55	13.29	1.01E-03
miR-34a-5p	3640.53	0.58	11.05	9.10E-03

TAA: thoracic aortic aneurysm. The traffic reflects the number of paths connected to one gene or one microRNA: the higher number of paths connected, the higher traffic value.

Table S8 – 79 TAA-associated genes predicted to be targeted by miR-574-5p – See Excel file.

ABL2	ACE2	ADAMTS10
ADAMTS14	ADAMTS5	ADRA1A
AKT2	ALPL	AXL
CD151	CDKN1A	CHST3
COL15A1	COL18A1	CREBBP
CTSD	CX3CL1	CX3CR1
DAG1	DDR1	DDX58
DPP4	EDN1	EDNRB
EFEMP1	ELANE	ELAVL1
FASLG	FGF18	FGFRL1
HTRA1	HYAL1	ICAM1
IGF1R	IL1RL1	ITGAL
JAM3	KITLG	LAMC3
LEP	MAPK1	MEF2C
MMP16	MMP3	MSN
NDRG4	NEDD4	NFAT5
NOD2	NPY2R	NR3C2
OLFML2A	OLR1	ORAI1
PAK1	PDGFB	PDGFRB
PGF	PIN1	PLEC
PPARA	PRKCA	PRKCE
PSEN1	PTGIS	ROCK2
S100A12	SEL1L	SERPINB9
SMAD2	SMAD4	SNAP23
SPRR3	TGM2	TIMP2
TRIB1	TTN	VEGFA
WNT1		

References

1. de Hoon, M. J.; Imoto, S.; Nolan, J.; Miyano, S., Open source clustering software. *Bioinformatics* **2004,** 20, (9), 1453-4.
2. Saldanha, A. J., Java Treeview--extensible visualization of microarray data. *Bioinformatics* **2004,** 20, (17), 3246-8.
